# Ancient DNA from 8400 Year-Old Çatalhöyük Wheat: Implications for the Origin of Neolithic Agriculture

**DOI:** 10.1371/journal.pone.0151974

**Published:** 2016-03-21

**Authors:** Hatice Bilgic, Erdogan E. Hakki, Anamika Pandey, Mohd. Kamran Khan, Mahinur S. Akkaya

**Affiliations:** 1 Department of Chemistry, Middle East Technical University, Cankaya, Ankara, Turkey; 2 Biotechnology Program, Middle East Technical University, Cankaya, Ankara, Turkey; 3 Department of Soil Sciences and Plant Nutrition, Selcuk University, Konya, Turkey; University at Buffalo, UNITED STATES

## Abstract

Human history was transformed with the advent of agriculture in the Fertile Crescent with wheat as one of the founding crops. Although the Fertile Crescent is renowned as the center of wheat domestication, archaeological studies have shown the crucial involvement of Çatalhöyük in this process. This site first gained attention during the 1961–65 excavations due to the recovery of primitive hexaploid wheat. However, despite the seeds being well preserved, a detailed archaeobotanical description of the samples is missing. In this article, we report on the DNA isolation, amplification and sequencing of ancient DNA of charred wheat grains from Çatalhöyük and other Turkish archaeological sites and the comparison of these wheat grains with contemporary wheat species including *T*. *monococcum*, *T*. *dicoccum*, *T*. *dicoccoides*, *T*. *durum* and *T*. *aestivum* at HMW *glutenin* protein loci. These ancient samples represent the oldest wheat sample sequenced to date and the first ancient wheat sample from the Middle East. Remarkably, the sequence analysis of the short DNA fragments preserved in seeds that are approximately 8400 years old showed that the Çatalhöyük wheat stock contained hexaploid wheat, which is similar to contemporary hexaploid wheat species including both naked (*T*. *aestivum*) and hulled (*T*. *spelta*) wheat. This suggests an early transitory state of hexaploid wheat agriculture from the Fertile Crescent towards Europe spanning present-day Turkey.

## Introduction

Even after several decades of research, wheat evolution and domestication remains a debate among ecologists, archaeologists and molecular breeders. Archaeobotanical records have shown that the Fertile Crescent played a crucial role in the advent of agriculture since it was the center of wheat domestication. However, new studies continue to reveal different aspects of wheat progression and encouraging people to pay attention to this huge region [1–10].

Wheat domestication began approximately 12000 years ago and it is considered a milestone in the development of human civilization [11, 12]; however, determining the initiation point of this domestication in the Fertile Crescent or elsewhere is controversial [13]. Several archaeological studies have shown the involvement of countries such as Korea, Spain and China in wheat domestication [9, 12, 14–18]. Scientists accredited the Karacadağ region close to Diyarbakir in southern Turkey with einkorn domestication [19] but the location of the first emmer domestication is widely debated [20–22]. Although the upper Jordan valley is believed to be the center of distribution of *T*. *dicoccoides* populations, Turkish populations have shown the allozyme based similarity with *T*. *dicoccoides* in population structure [23]. Several biomolecular and archaeological studies have been carried out in order to investigate the primitive Turkish wheat attained from different archaeological sites.

Çatalhöyük is a huge Neolithic archaeological settlement situated on the route to Europe in central Turkey dating back to 7400–6000 BC (calibrated). Previously, it was assumed that there were no Neolithic settlements in Anatolia due to cold weather conditions [24, 25]. Contrary to such assumption, the discovery of Çatalhöyük by the British archaeologist James Mellaart in 1952 and its excavation during the period 1961–1964 made it an internationally recognized archaeological site [11, 26, 27]. One excellent finding was the charred grains of near east originated hexaploid wheat [28]. Not only were the seeds very accurately dated and the excavation locations precisely recorded, but also they were well preserved in relation to other recoveries from ancient world [28, 29]. However, the detailed archaeobotanical description of the samples is still lacking. The discovery of charred hexaploid wheat grains in Çatalhöyük crucially questioned the relation with primitive or contemporary wheat forms; thus, we decided to analyze the samples of the unusually well preserved charred Çatalhöyük wheat.

Ancient DNA analysis is an interdisciplinary area of research utilizing molecular biological techniques to investigate archaeological questions and find hidden clues. Ancient DNA is a distinctive source in the study of the genetic constitution of biological remains from archaeological excavations. Even a tiny DNA fragment can be used to genetically identify different wheat species, thus allowing the stages of wheat domestication to be presented in dimensions of time and space. Charred wheat seeds are an efficient source of ancient DNA assessment because of their good state of preservation. Thus, archaeobotanical analyses of charred wheat seeds contributed extensively to the existing knowledge of wheat domestication and its spread. Although there are several studies on ancient wheat DNA from different archaeological sites, there are still unanswered questions related to wheat domestication and the exploitation of ancient DNA methodology is still promising for revealing how wheat was domesticated.

To date, a number of researchers have compared partial sequences of high molecular weight (HMW) subunit genes of glutenin protein in ancient wheat DNA studies. These proteins in wheat are important in developing bread-making quality. HMW subunit genes are capable of successfully identifying the ploidy level of primitive and wild wheat seeds due to their multi-allelic and sub-genome specific nature. Partial sequence comparisons revealed biogeographical distributions of glutenin allele lineages in 3000-year-old wheat DNA from Assiros-Greece and modern wheat samples [30]. Schlumbaum, et al. [31] used a glutenin promoter region of ancient wheat DNA to distinguish tetraploid and hexaploid charred wheat seeds recovered in Switzerland. Blatter, et al. [32] identified spelt specific alleles from 300-year-old spelt spikelets in Switzerland and presented a discussion on the European origin of spelt. Fernández, et al. [9] undertook ancient wheat DNA analysis on charred grains of naked wheat and barley from several archaeological sites in Spain. The findings of these studies contributed to the existing knowledge of the agricultural evolution of European wheat. In the current study, we focused on the origin of wheat domestication under spatial and temporal dimensions using DNA analysis of Çatalhöyük stock and samples retrieved from other archaeological sites in Turkey.

There are several obstacles in ancient DNA-based research that include less recovery of DNA during isolation; frequent contamination by microbial or fungal DNA; the risk of contamination with modern plant DNA, especially during Polymerase Chain Reaction (PCR); the fragmented nature of ancient DNA; reduced efficiency in PCR and the amplification of relatively short ancient DNA fragments [33–36]. In addition, there are the specific requirements of experimental factors meeting the authenticity criteria of isolated DNA [37–39] including controls for both extraction and PCR amplification reactions; dedicated laboratory facilities; and obtaining reproducible results in different laboratories. Moreover, it is important to repeat amplification, cloning and sequencing of same extract as this may lead to the recovery of novel alleles that are unnoticed in modern species.

**Fig 1:**
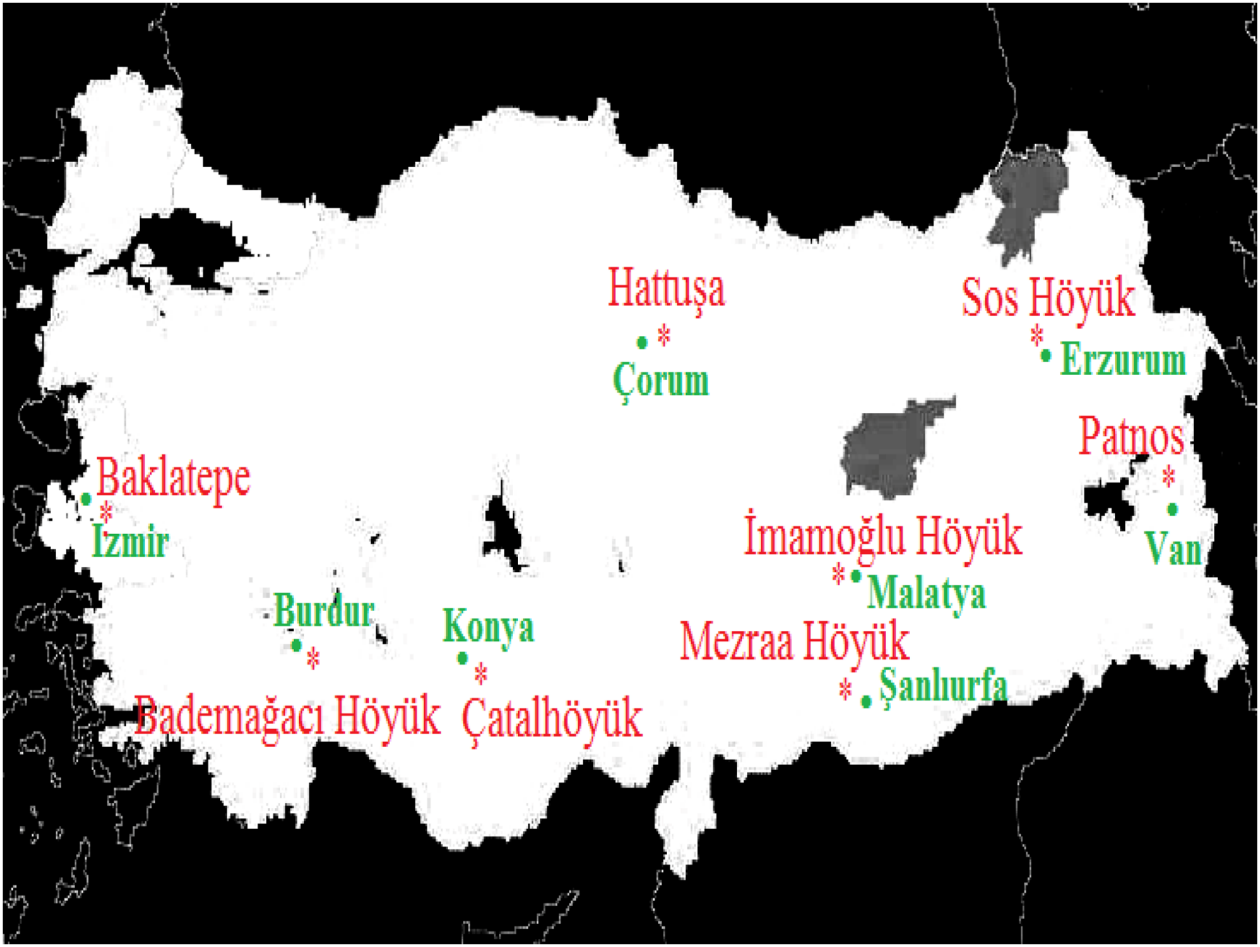
Locations of the Turkish archaeological sites. Locations of the Turkish archaeological sites where the ancient wheat samples were obtained. [Image is for representative purpose only. [Source—http://sedac.ciesin.columbia.edu/gpw. Licensed under Creative Commons 3.0 Attribution License.]

In this study, we describe the isolation, amplification and sequencing of short DNA fragments from charred Çatalhöyük wheat grains. The seeds were first characterized as einkorn and emmer wheat at the excavation sites and estimated to date from 6400 and 6200 BC (calibrated), respectively [40]. This is the first ancient wheat DNA report from the Middle East and describes the recovery of the oldest wheat DNA to date using PCR methodology in accordance with the authenticity criteria [35]. We were able to carry out the labeling of PCR products with high authenticity by incorporating the radiolabeled nucleotide allowing the product size and appearance to be clear and observable on the denaturing DNA sequencing gel-autoradiograph. Aiming to recover ancient wheat DNA and phylogenetically characterize the ancient wheat species, this study is not only based on the most ancient archaeological wheat DNA at molecular level, but it is also the first genetic study of wheat remains from Turkish archaeological sites (Fig 1). The results of this work considering the temporal and spatial dimensions will provide the supportive DNA based evidence to contribute to the existing wheat evolution and domestication studies.

## Materials and Methods

### Archaeological wheat samples

**Fig 2:**
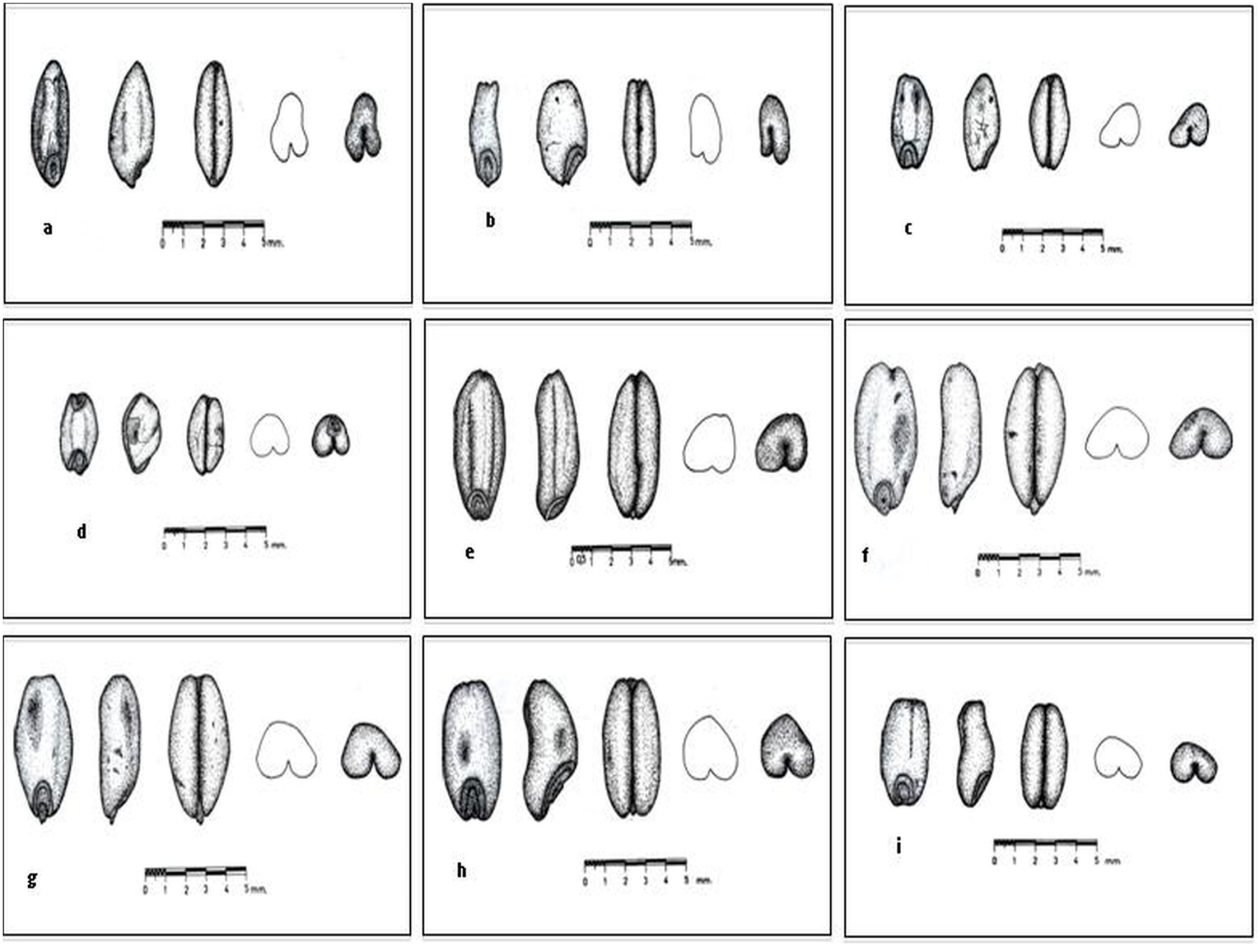
The hand drawn pictures of ancient seeds from Çatalhöyük and Imamogğlu Höyük. a) Typical einkorn; hulled domesticated diploid wheat (*T*. *monococcum*: identified by EOD & DM); A single seed from Çatalhöyük, originally labeled as an einkorn sample (*T*. *monococcum*) (EVI.17, 1962. b) Typical einkorn; hulled domesticated diploid wheat (*T*. *monococcum*; identified by EOD & DM); A single seed from Çatalhöyük, originally labeled as an einkorn sample (*T*. *monococcum*) (EVI.17, 1962). c) Atypical einkorn; a transition type between einkorn (diploid) and naked wheat (tetra/hexaploid). A single seed from the Çatalhöyük62 sample (*T*. *monococcum*) (EVI.17, 1962), identified by D. Martinoli. d) Naked wheat; tetraploid or hexaploid free treshing wheat (*T*. *durum/aestivum*). A single seed from the Çatalhöyük62 sample (*T*. *monococcum*) (EVI.17, 1962), identified by D. Martinoli. e) Typical emmer; hulled domesticated tetraploid wheat (*T*. *dicoccum*; identified by DM); A single seed from Çatalhöyük, originally labelled as an emmer sample, E IV.4, 1961 (*T*. *dicoccum*). f) Atypical emmer; a transition type between emmer (tetraploid) and naked wheat (tetra/hexaploid: identified by DM); A single seed from Çatalhöyük originally labeled as an emmer sample E IV.4, 1961 (*T*. *dicoccum*) g) Naked wheat; tetraploid or hexaploid free threshing wheat (*T*. *durum/T*. *aestivum*; identified by DM); A single seed from Çatalhöyük, originally labeled as an emmer sample E IV.4, 1961 (*T*. *dicoccum*). h) Naked wheat; tetraploid or hexaploid free threshing wheat (*T*. *durum/T*. *aestivum*; identified by EOD); A single seed from the İmamoğlu Höyük sample. i) Naked wheat; tetraploid or hexaploid free threshing wheat (*T*. *durum/T*. *aestivum*; identified by EOD); A single seed from the Patnos sample. EOD: E. O. Dönmez of Department of Biology, Haccettepe University and DM: D. Martinoli, Swiss Biodiversity Forum, Switzerland, Botany (drawn by a commercial graphic artist and further cross-checked by the scientists involved in the study).

The charred wheat seed samples used in this study were obtained from different Turkish archaeological sites and ranging from Neolithic to Hellenistic periods (~6400 BC to ~700 BC) (Table 1, Fig 1). The archaeological wheat samples from Çatalhöyük and İmamoğlu Höyük were given by Professors Ay Melek Özer and Şahinde Demirci from the Department of Archaeometry, Middle East Technical University, Turkey; Gordon Hillman from the Department of Archaeology, UCL, UK; and Emel Oybak Dönmez from Department of Biology, Hacettepe University, Turkey. While other archaeological samples were given by the Turkish Ministry of Culture, Monuments and Museums, General Directorate in Turkey and the heads of excavations at Bademağacı Höyük (Prof. Refik Duru, Department of Archaeology, Istanbul University), Baklatepe (Prof. Hayat Erkanal), Hattuşa (Prof. Jurgen Seeher), Sos Höyük (Prof. Tony Sagona), Mezraa Höyük (Assoc. Prof. Tuba Ökse) and İlhan Temizsoy from the Ankara Anatolian Civilizations Museum in Turkey. The locations and excavation levels of these samples are presented in Table 1. As several archaeobotanists working at the Çatalhöyük excavation site emphasized on the abundance of hexaploid wheat at Catalhoyuk Site, because the distinctive chaff (rachis) occurs frequently [41], it was extremely crucial to collect the ancient seed samples with utmost care. Hence, the Çatalhöyük61 and Çatalhöyük62 samples were crudely labelled and bagged at the excavation sites by the excavation heads and designated as einkorn and emmer wheat, respectively. However, as the ancient wheat seed samples were found in clay containers, there was no chance of contamination. Apart from those from Çatalhöyük, the other samples were dated based on archaeological evidence. The dating of the Çatalhöyük samples was conducted using calibrated ^14^ C, dendrochronology and wiggle match methods as well as plaster counts [26, 29]. We chose representative seeds with spherical, ellipse and oval shapes for DNA isolation and amplification (Fig 2).

**Table 1:** Archaeological wheat samples analyzed in this study.

Sample	Location	Excavation level	Dating	Period	Species abrv.	DNA ext.	PCR
Çatalhöyük62	Konya	CH62 EVI.17 Bin7	6400 BC	Neolithic	*Tm* (E) */ Tsp*	6	+
Çatalhöyük61	Konya	CH61 EIV.4	6200 BC	Neolithic	*Tdc* (M) */ Tsp*.	8	+
Bademağacı Höyük98	Burdur	Neo3 and Neo4	5000 BC	Neolithic	*Tdc / Tdm / Ta*	2	-
Baklatepe96	İzmir	BT96 H-15;VIII-X/d-j; 58.22–58.14 cm	4000 BC	Late Chalcolithic	*Tdc*	1	-
Sos Höyük, 1999	Erzurum	8/8 L17b 4299 s.212	3500–3000 BC	Late Chalcolithic	*Tdm / Ta*	1	-
Sos Höyük, 2000	Erzurum	M17 3769 s.130	3500–3000 BC	Late Chalcolithic	*Tdm / Ta*	1	-
İmamoğlu Höyük86	Malatya	Not known	2300–2000 BC	Early Bronze Age	*Tdm / Ta*	15	+
Hattuşa99	Çorum	311/342.66	1400–1300 BC	Bronze Age	*Tm*	2	-
Patnos61	Van	Not known	800–700 BC	Urartu	*Tdc / Tdm / Ta*	13	+
Mezraa Höyük2000	Şanlıurfa	Çukur D	300–700 AD	Hellenistic	*Tdm / Ta*	2	-

*Tm*: *T*. *monococcum*, *Tsp*: *Triticum species*, *Tdc*: *T*. *dicoccum*, *Tdm*: *T*. *durum*, *Ta*: *T*. *aestivum*. The numbers on the right of the sample names denote the date of excavation that yielded that particular wheat sample. Patnos wheat was a museum specimen for which exact recovery date could not be determined, the other dates were provided by the excavation heads or the archaeobotanists. The species name follows archaeobotanical identification; for the Çatalhöyük samples, it is a combination of original labeling and archaeobotanical identification: E for einkorn, M for emmer. DNA ext. denotes the total number of DNA extractions performed using different methods. Positive PCRs were obtained at HMW glutenin loci. The excavation level corresponds to natural strata at the time of the systematic digging of archaeological site. These levels are counted in increasing order from top to bottom.

### Modern wheat samples

In order to make an efficient genetic comparison with ancient wheat at glutenin loci, we included 26 bread, durum and wild wheat genotypes together with progenitor species in the experiment (S1 Table).

### DNA extraction

To achieve authenticity, all the DNA extractions and PCR reactions were performed in two different physically isolated laboratories at University of Manchester, UK (Brown Lab) and Middle East Technical University, Turkey (Akkaya Lab), respectively. Dedicated equipment was used for the analysis of the archaeological material to prevent contamination with modern DNA. Several methods were trialed for the ancient wheat DNA isolations [42–44] including a modified protocol to the method of Rogers and Bendich [45] by Allaby et al. [30], which was found to be most promising procedure. For the DNA extraction, 0.5 g charred seeds were crushed and 750 μL of Buffer containing 2% w/v CTAB together with 100 mM EDTA pH 8.0, 20 mM Tris-Cl pH 8.0 and 1.4 M NaCl was added. An extraction blank was also assembled. The samples were incubated at 60°C for 1 hour in water bath followed by centrifugation at 14000 rpm for 10 min. To the obtained supernatant, 500 μL of chloroform:isoamyl alcohol (24:1) was added and the mixture was again centrifuged at 14000 rpm for 2 minutes. The aqueous supernatant containing DNA was carefully collected and double volume of Buffer 2 including 1% w/v CTAB (Cethyl trimethyl ammonium bromide), 50 mM EDTA pH 8.0, 10 mM Tris-Cl pH 8.0 was added. After overnight incubation at 4°C, the extract was centrifuged for 20 min at 14000 rpm for the DNA precipitation and the resulting pellet was re-suspended in 50 μL of double distilled water. Five molar NaCl and 100% ethanol were added to the re-suspended pellet in 0.2 volume and 4 volume, respectively. The mixture was incubated at –20°C for 6–12 h and then centrifuged at 14000 rpm for 20 min. The DNA pellet was re-suspended in 50 μL sterile dd H_2_O and stored at -20°C. Since during extraction, there is a possibility of contamination of the foreign DNA such as fungi and bacteria physically attached to the seeds, electroelution was followed by ethanol precipitation. The DNA isolation for the modern wheat samples was performed in a different laboratory space in the Akkaya Lab, Middle East Technical University, Turkey according to the method adopted by Saghai-Maroof, et al. [44].

### PCR Amplification

The majority of PCR amplifications were directed at the HMW glutenin partial promoter region with three primer sets targeting at 241–243 bp, 152–156 bp and 106–107 bp in length. The main primers were designed to amplify the upstream to the open reading frame of the HMW glutenin subunit gene. However, two different types of 152–156 bp target primer sets, Glu_156_A/B and Glu_156_D, designed on the A/B and D genome copies of the glutenin protein, respectively, were used in the study (Table 2). The PCR reaction mixture contained 5 μL of DNA extract, 5–20 ng of each of the forward and reverse primers, 0.2 mM dNTP mix (2.0 mM each), 1.5 mM MgCl_2_, 1X PCR Buffer (10 mM Tris-HCl, pH 8.3; 50 mM KCl), 1.5 units Taq DNA polymerase and 0.04–0.10 μL (depending on the half-life) of ∝^32^P-dATP (3000 mCi/mmol) for radioactive labelling. The applied PCR conditions were 30–35 cycles of (94°C 2 min., 60°C 1 min., 74°C 1 min.) followed by a final extension at 74°C for 8–10 min., The annealing temperature was 58°C for 156 and 107 bp length target PCR reactions. Negative controls, without DNA, were used in PCR reactions to detect contamination. The radio-labeled PCR products were visualized on 6% DNA denaturing sequencing polyacrylamide gels. Radioactive labeled DNA run on gels can be directly utilized to obtain auto-radiographic image. The density of the band images can be employed to determine the relative quantities of the radio-labeled DNA in the sample.

**Table 2:** Target regions Nuclear-HMW glutenin promoter and sequences of the PCR primers utilized in the ancient DNA amplifications in the current study.

PCR Primers	Sequence (5’→3’)
Glu_243Fwd	GATTACGTGGCTTTAGCAGAC
Glu_243Rev	TGCTCGGTGTTGTGGGTGAT
Glu_156_DFwd	CAAAGCTCCAATTGCTCCT
Glu_156_DRev	TTTATAGGGACGTGGTGAAG
Glu_156_A/BFwd	CAAAGCACCAATTGCTCCT
Glu_156_A/BRev	TTTATAGGGACGAGGTGAAG
Glu_107_A/BFwd	GCTTYTTTTGTGTTGGCAAAYT
Glu_107_A/BRev	GTTCRKGACMATGGYTGYGT

The degenerate bases in primer sequences stand are as follows: Y:C/T; R:A/G; K:G/T; M:A/C

### Cloning of PCR products and sequencing

The bands at the expected sizes for all the wheat samples on agarose gel and Çatalhöyük samples on denaturing DNA sequencing polyacrylamide gels [46] were cut by a sterile lancet for DNA extraction by freezing and thawing in liquid nitrogen. The extracted DNA was cloned into pGEM T-Easy vector (Promega) and the transformed *E*. *coli*, DH5α or JM109 with the ligation products were spread onto LB-ampicillin plates. Recombinant clones were selected based on the inactivation of the lacZ' gene by blue-white selection. Furthermore, mini plasmid isolation was conducted and inserts were custom sequenced at the Keck Biotechnology Resource Laboratory of Yale University.

### Phylogenetic analysis of the sequences

The ancient and modern wheat DNA sequences obtained for glutenin loci were aligned by multiple alignments in ClustalX 1.8 program and dendrograms were constructed using the Neighbor-Joining Method according to the length of the sequences. The Blastn algorithm was employed to determine homologous sequences for the obtained sequences that were consequently aligned at ~100 bp, ~150bp and ~250bp length, respectively. Randomly selected ancient and modern wheat DNA sequences available from GenBank were also included in the phylogenetic analysis undertaken in our study.

## Results

It is difficult to extract amplifiable DNA from archaeological wheat samples due to their having been exposed to diverse environmental conditions for centuries. The amount of starting material for extraction, age of archaeological samples and state of preservation of the archaeological seeds are some of the crucial factors affecting both the quality and quantity of DNA and its amplification [35, 36]. Based on these factors, we found PCR products of different intensity in our experiments (S1 and S2 Tables). For older samples, an increase in the amount of starting material was required as was the case for the Çatalhöyük62 samples, since 0.5 g of seeds did not reveal DNA; however, 1.5 g of seeds extracted with the same method did contain DNA, which was amplified by PCR (S2 Table).

The state of preservation of the archaeological seeds also has an equal effect on the DNA extraction. The youngest archaeological wheat sample from Hellenistic level of Mezraa Höyük did not yield amplifiable DNA in two extracts both obtained with 0.5 g starting material, this was possibly due to the poor preservation state of the seeds (S2 Table). On the other hand, two different sets of Patnos samples used in our study, namely emmer and naked wheat with black and bright grey colored charred seeds, respectively, showed an altered response. The Patnos emmer wheat samples provided amplifiable DNA with 0.5 g and 1.5 g sample as starting material, whereas the Patnos naked wheat samples provided amplifiable DNA with neither the 0.5 g sample nor even with 20 seeds sample as starting material. On the other hand, the Hattusa samples were intensively black colored and well preserved, but did not yield amplifiable DNA may be due to limited number (2) of extracts.

As expected, repeated extractions performed per sample increased the probability of obtaining amplifiable ancient DNA (Table 1). Except for the Çatalhöyük61, Patnos and İmamoğlu Höyük samples, the stocks available were limited, thus only 1 to 2 extractions were performed with 0.08 to 0.5 g starting material, none of which revealed amplifiable DNA.

**Fig 3:**
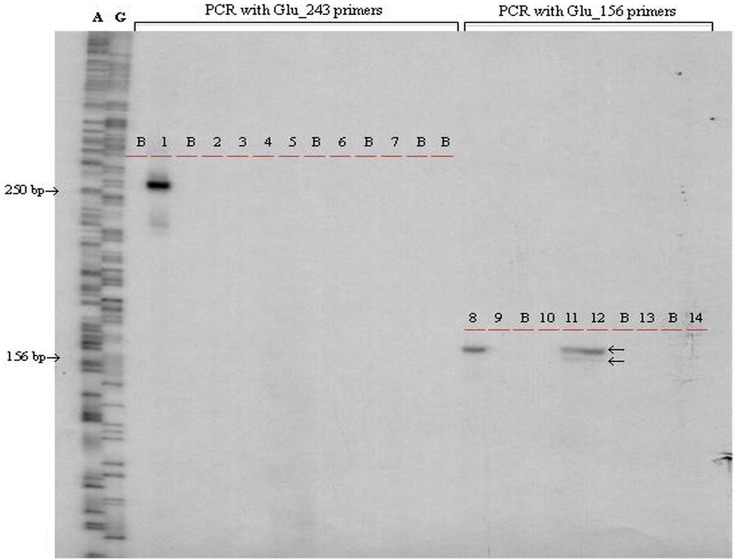
Autoradiograph of the radioactively labeled PCR amplification products of the Çatalhöyük samples separated on DNA sequencing gel. 1) Modern *T*. *durum* targeting 243 bp long PCR amplification product of Glu locus. 2) No DNA, negative control of PCR loaded on lane 1. 3) PCR with Çatalhöyük samples extraction blank. 4) PCR with Çatalhöyük62 and 5) Emmer DNA isolates. 6) PCR with Baklatepe sample extraction blank. 7) PCR with a Baklatepe sample. 8) Modern *T*. *durum*. 9) No DNA, negative control of PCR loaded on lane 8. 10) PCR with extraction blank. 11) PCR with Çatalhöyük62 and 12) Çatalhöyük61 DNA isolates. 13) PCR with a Baklatepe sample extraction blank. 14) PCR with a Baklatepe sample. B) Blank lanes. Lanes A) DNA sequencing reaction products with ddATP and G) DNA are sequencing reaction products with ddGTP of M13mp18 ssDNA using a T7 primer. The arrows on the left indicate the lengths in bp, the arrows on the right (lanes 11 and 12) indicate the top and bottom alleles in the Çatalhöyük samples.

In the PCR reactions, two Çatalhöyük samples, one Imamoğlu and one Patnos sample gave successful and reproducible amplification at HMW glutenin loci (Table 1, Fig 3). The PCR reactions of the Çatalhöyük samples targeting the ~250 bp region of the HMW glutenin loci did not produce an amplicon, however, those targeting 150 and 100 bp region resulted in clonable PCR products. There was no contamination with the modern wheat DNA which can be considered as an evidence for the authenticity of the samples. For the Çatalhöyük61 samples, ~250 bp portion of the glutenin partial gene failed to be amplified by PCR, while ~150 bp and ~100 bp partial promoter sequences of the HMW glutenin protein were amplified. Two different extracts from the Çatalhöyük61 samples and a single extract from the Çatalhöyük62 sample were used to derive sequences from three and two separate PCRs, respectively. For Çatalhöyük62, the first and second extracts were amplified with Glu_156_D, Glu_156_A/B and Glu_107 primers, respectively. Bands of radio-labeled PCR products were isolated from denaturing DNA sequencing gels and further, cloned and sequenced (S1–S4 Figs). The same region of 26 modern einkorn, emmer, durum and bread wheat samples from Turkey were also cloned and sequenced after amplification with either Glu_243 or Glu_156_D and Glu_156_A/B. We obtained a total of 48 modern wheat sequences from 26 different samples (S3 Table). The DNA sequences obtained from the Çatalhöyük samples revealed A, B and D genome copies of glutenin protein. No jumping PCR phenomena or disturbed sequences were observed. A total of 22 sequences were at 152–156 bp length bearing 17 alleles of which 13 were new to GenBank, while 10 sequences were at 103–107 bp length bearing five different alleles of which two were new to GenBank. It was noteworthy to obtain D genome copies of the HMW glutenin in both the Çatalhöyük61 and Çatalhöyük62 samples since they had previously been labeled as einkorn (diploid, AA genome) and emmer (tetraploid, AABB genome) by morphological criteria applicable to charred wheat seeds.

İmamoğlu Höyük wheat was amplified with all primer sets for the ~250, ~150 and ~100 bp partial promoter sequence of HMW glutenin protein from 5 different extracts, while Patnos wheat (*T*. *dicoccum*) was amplified with primer sets for the ~250 and ~150 bp partial promoter sequence of HMW glutenin gene alleles from 3 different extracts (S3–S5 Figs). Unfortunately, in the Patnos samples unfortunately cloning failed. In the İmamoğlu Höyük naked wheat samples 16 sequences contained A, B and D genome copies of glutenin alleles from five different PCR products of four different DNA extracts. Eleven, 2 and 3 İmamoğlu Höyük sequences were obtained at ~100, ~150 and ~250 bp length, respectively. Of these, two ~150 bp and two ~250 bp long sequences were different from all the known HMW partial glutenin alleles and were deposited in GenBank. A total of 32 Çatalhöyük, 16 İmamoğlu Höyük and 48 modern wheat sequences were obtained at glutenin loci and aligned in accordance with their length by ClustalX 1.8 software after excising the primer sites. Sequence alignments of ~100 bp, ~150 bp and ~250 bp groups contained 21, 43 and 32 sequences, respectively. A consensus sequence for each based on 80% similarity is given as a last entry in the multiple alignments.

ClustalX 1.8 constructed different Neighbor-Joining (NJ) trees, after excising primer sites. All the trees were bootstrapped 1000 times and NJ dendograms were drawn based on ~100 bp, ~150 bp and ~250 bp sequences. At ~100 bp length, the majority of Çatalhöyük and İmamoğlu Höyük sequences were identical (S6 Fig). However, the variation and novelty of the ancient DNA sequences became visible at 150 bp (S7 Fig). At both the GluA1 and GluB1 alleles, the Çatalhöyük sequences were significantly more diverse than modern wheat sequences. At the GluB1-1 locus, modern wheat samples shared the same allele, while ancient wheat sequences differed from each other. In addition, wild progenitors from Diyarbakır Karacadağ, KCD12ddes1 and KCD12ddes2 were found to be greatly similar to each other as well as to modern durum and bread wheat cultivars. At the GluA1-1 locus, *T*. *monococcum* and *T*. *boeoticum* clustered separately from modern wheat alleles demonstrating the close relationship between the two A genome diploids. Interestingly, at the same locus, cultivated einkorn from Kastamonu shared one allele with wild einkorn from Karacadağ.

The NJ tree based on ~250 bp long sequences (S8 Fig) mostly contained modern sequences and three ancient sequences from the İmamoğlu Höyük samples, which clustered with the cultivated tetraploid wheat (*T*. *dicoccum*) and not with einkorn and wild emmer wheat at the Glu A1-2 and Glu B1-1. At the GluA1-2, the wild and cultivated einkorn samples from western Turkey (Balıkesir) were clustered together. Wild einkorn samples clustered distantly from polyploids and diploids while the cultivated form was grouped with wild einkorn samples from Karacadağ and Şanlıurfa.

Also at the GluA1-1 locus (S8 Fig), wild einkorn wheat (*T*. *urartu*) was found distant from polyploid wheat. However, diploid *T*. *monococcum* sample from Ağrı was found misclassified as it grouped with polyploid wheat allele at the glutenin loci. A similar misclassification at both ploidy and species was observed in our microsatellite markers based study and that sample was excluded from the remainder of the ancient wheat DNA analysis. At the GluB1-2 locus, cultivated emmer from Turkey was strongly associated with wild emmer from Karacadağ (S8 Fig). On the other hand, at the GluD1-2 locus, *Aegilops tauschii* from Şanlıurfa, clustered separately from cultivated bread wheat (*T*. *aestivum*), although it is considered to be the wild progenitor of the D genome in hexaploid wheat species.

To determine the genetic similarity among all the modern and ancient wheat samples, longer sequences were excised according to the shortest i.e. 107 bp ancient fragment and then aligned (S9 Fig). The combined dendrogram was in agreement with the information provided in individual dendrograms. In combined tree at the GluA1-1 loci, *T*. *urartu* sequences were clustered with polyploid wheat supporting the concept of *T*. *urartu* as progenitor of the A genome of tetraploid and hexaploid wheat.

**Fig 4:**
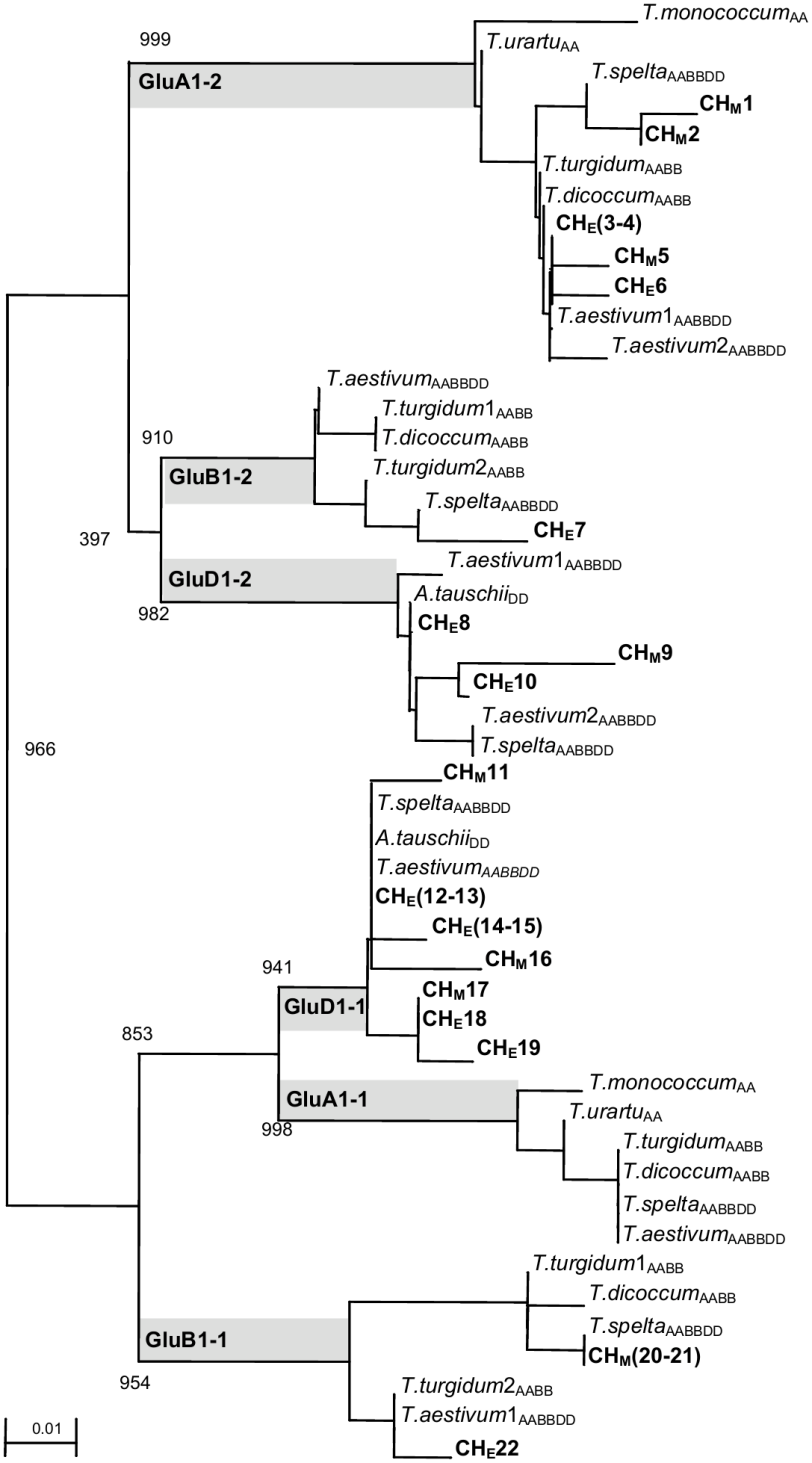
A Neighbor-Joining tree showing the genetic similarity between Çatalhöyük (CH) and contemporary wheat species (*Triticum* sp.). **A** Neighbor-Joining tree showing the genetic similarity between the Çatalhöyük (CH) and contemporary wheat species (*Triticum* sp.) based on the DNA sequences excluding the PCR primer sites. CH_M_ (Çatalhöyük61) and CH_E_ (Çatalhöyük62) denote the sequences obtained from samples previously classified as emmer (CH61 E.IV, ~6200 BC_calibrated_) and einkorn (CH62 E.VI, ~6400 BC_calibrated_), respectively; parentheses denote multiple clone copies of the same allele. Genomic compositions are presented as the subscript to each species, including two hexaploid forms, naked (*T*. *aestivum*) and hulled (*T*. *spelta*) wheat. The Bootstrap values are printed next to the branches. The sequences have been deposited in to GenBank under the accession numbers AF528823-AF528844.

The Çatalhöyük samples amplified at ~150 bp length were compared to glutenin sequences available in GenBank using the Blastn search and a NJ tree was constructed to determine their similarity with contemporary wheat species (Fig 4). D genome alleles were found in the Çatalhöyük samples that were originally classified as diploid and tetraploid species. The Blastn search of GenBank revealed that the majority of A genome alleles were similar to naked wheat (durum and bread wheat) rather than the AA genome diploids (*T*. *boeticum*, *T*. *monococcum*, *T*. *urartu*). Interestingly, some of the A genome alleles and many B genome alleles were most similar to spelt wheat from Europe at 152–156 bp length. Using the same methodology, the genetic relationship between the Imamoğlu Höyük sequences and modern wheat from Turkey and GenBank, respectively was determined (S10 Fig). The majority of sequences from the İmamoğlu Höyük samples were more similar to A, B & D genome copies of modern hexaploid wheat, two were most similar to cultivated tetraploid species (*T*. *turgidum*) and interestingly, one sequence amplified at the B1-1 locus (IM17) was most similar to hulled hexaploid wheat species, *T*. *spelta* at its full length of ~150 bp.

## Discussion

Since most archaeological samples are charred and exposed to different environmental conditions that affects both the quality and content of DNA, it is difficult to extract amplifiable DNA. Our results reveal the possibility of increasing the amplifiable amounts of DNA by increasing the amount of starting material (S2 Table). Both the Çatalhöyük61 and Çatalhöyük62 samples provided amplifiable DNA by increasing the initial seed material from 0.5 g to 1.5 g. The age of archaeological samples is a major factor in the success of ancient DNA extractions. In our study, the well-preserved state of Çatalhöyük samples allowed the generation of short but relevant PCR products. The youngest archaeological wheat sample from Hellenistic level of Mezraa Höyük was considered to be the most probable to contain amplifiable DNA in terms of age. However, it was collected from just below the soil surface of Höyük (E. Oybak Dönmez, pers. comm.) and might have been exposed to a variety of environmental eroding factors (such as sun, rain, wind and frost). Additionally, although carbonization may preserve the seeds to a certain degree, the charred seeds from Mezraa Höyük were substantially deformed due to both the charring process and being attacked by insects leaving obvious boreholes in the seeds. As a result, due to the very poor preservation state of the material, it did not yield amplifiable DNA (S2 Table).

The color of charred seeds reflects the intensity of the temperature to which they have been exposed during the charring process [47]. Grey-colored charred seeds are supposed to have borne a higher charring temperature with more damaged DNA in comparison to black-colored seeds. In the current study, the black-colored Patnos emmer wheat samples were founded to yield DNA supporting the idea that exposure to eroding environmental conditions, infestation by insects and the temperature during charring determines the extent of DNA survival.

DNA extraction from old archaeological samples may create some problems in PCR amplifications. Even if DNA is present, the amount of amplifiable DNA may be very low. In addition, in our study, instead of long fragments that are required for amplification, only small pieces of nucleotides were generally obtained; however, we succeeded in achieving amplifiable DNA from the samples using alternative shorter and less elaborate DNA extraction methods.

During DNA amplification, HMW glutenin primers provided reproducible amplification results in the expected range of 241–243 bp based on the genome copies. Primers for the high molecular weight (HMW) glutenin subunit gene were designed and internal glutenin primers for the shorter pieces (156 bp and 107 bp-degenerate) of the targeted region. These primers aimed to prove the authenticity of the amplification of the glutenin locus and to amplify the shorter ancient DNA pieces.

Although, the wheat samples from Çatalhöyük used in this study were previously labeled as diploid (einkorn) and tetraploid (emmer) (personal communication of H. Bilgic with G. Hillman), a more detailed archaeobotanical analysis (D. Martinoli) revealed that both samples might contain a small amount of atypical or naked wheat grains (Fig 2) thus emphasizing the necessity of genetic analysis. The ~250 bp portion of the glutenin partial gene was not amplified by PCR providing evidence of the ancient origin of the DNA extracts from Çatalhöyük. The absence of jumping PCR phenomena or disturbed sequences suggested that the Çatalhöyük samples had good quality DNA. The retrieval of D genome glutenin copies of the hexaploid wheat from Çatalhöyük62 and Çatalhöyük61 sequences demonstrates the power of the DNA-based method to distinguish the ploidy levels to justify the species. The study provided the genetic evidence that the sample collection contained hexaploid wheat (AABBDD) from an early Neolithic period in central Turkey.

From the Çatalhöyük samples, 15 novel sequences were deposited in GenBank however, only four new alleles were recovered from the samples from the İmamoğlu Höyük site. The older the samples were, the higher the incidence of new alleles at the glutenin loci. These results were in accordance with the ages of ancient wheat samples reflecting the longer time span in the evolution of wheat.

In phylogenetic analysis, at the GluB1-1 locus, the modern wheat samples and wild progenitors were more similar to each other in comparison to the ancient wheat reflecting a greater genetic diversity among ancient wheat sequences. In addition, the adjacent clustering of *T*. *monococcum* and *T*. *boeoticum* at the GluA1-1 locus justified the close association between the two A genome diploids. At the same locus, the high genetic similarity of Karacadağ wild samples to cultivated einkorn from Kastamonu confirmed the proposal of Heun et al [19] about Karacadağ being the location of einkorn domestication (S7 Fig).

The grouping of the İmamoğlu Höyük samples with cultivated tetraploid wheat (*T*. *dicoccum*) at the GluA1-2 and GluB1-1 loci validated the archaeobotanical classification of the İmamoğlu Höyük sample as naked polyploid wheat (*T*. *durum/aestivum*) (S8 Fig). According to Heun, et al. [19], two subspecies of wild einkorn are distributed in the western (including Balkans) and eastern Turkey (including southern Anatolia and Fertile Crescent), respectively. The western wheat form, sp. *aegilopoides* is supposed to be distant from the cultivated einkorn, while the eastern wheat form *sp*. *thoudar* is genetically found to be the progenitor of cultivated einkorn. This was supported by our results in which at the GluA1-2 locus, the wild einkorn sample from western Turkey (Balıkesir), were distant from the clusters of polyploids and diploids, while the cultivated einkorn (*T*. *monococcum*) clustered with the wild einkorn samples from Karacadağ and Şanlıurfa. The clustering of *T*. *urartu* sequences with polyploid wheat and not with *T*. *monococcum and T*. *boeoticum* at the GluA1-1 loci in the combined dendrogram of modern and ancient wheat samples at 107 bp again supported the proposal that *T*. *urartu* was the progenitor of the A genome for tetraploid and hexaploid wheat (S9 Fig).

As stated, obtaining D genome alleles in the Çatalhöyük samples demonstrated the cultivation of hexaploid wheat in Çatalhöyük during the 7^th^ millennium BC. In addition, during a Blastn search at GenBank, the majority of A genome alleles in the Çatalhöyük sequences were found to be similar to naked wheat (durum and bread wheat) rather than AA genome diploids (*T*. *boeticum*, *T*. *monococcum*, *T*. *urartu*). This was additional evidence for the incidence of hexaploid wheat among the Çatalhöyük samples (Fig 4).

The similarity of A and many B genome alleles with spelt wheat at 152–156 bp length should also be considered. Spelt is a hexaploid hulled wheat frequently encountered in Europe since the Bronze Age, but absent in Near East records, except the indefinite and rare Neolithic incidents from Erbaba in central Turkey and Yarim Tepe in Northern Iraq (Figs 1 and 4). Our study provided the first genetic evidence for the possible presence of spelt wheat in the Near East and particularly in Turkey during the Neolithic age. It is a crucial finding that may assist in solving the mystery of the origin of spelt wheat. However, since there is no archaeobotanical evidence for spelt wheat cultivation, deposition or utilization from Turkish archaeological sites, the findings can be interpreted in two ways. First, there was a spontaneous occurrence and cultivation of spelt wheat or similar species mixed with einkorn, emmer and hexaploid wheat. Second, that primitive einkorn, emmer and bread wheat contained transitory forms resembling spelt due to absence of definite species barriers. Such intermediate forms of spelt-similar emmer wheat were observed in East Anatolia by Hillman, et al. [27]. Moreover, the relationship of the Imamoğlu Höyük samples to spelt wheat appear to reflect the accidental occurrence of an intermediate species among cultivated naked wheat rather than the true cultivation of spelt wheat at İmamoğlu Höyük. If the spelt-like wheat of Çatalhöyük and İmamoğlu Höyük are assumed to represent a general agriculture trend of its time, the results suggested a tremendous decrease in the cultivation of spelt-like wheat species in Anatolia over 4000 years. The discussions above can be regarded as speculative, due to the limited number of sequences and that they belong to a single nuclear glutenin locus. Being a promoter region, the HMW glutenin locus limits the collected polymorphism content and may not provide sufficient phylogenetic resolution. In addition, insufficient DNA in the samples and insufficient numbers of sequenced clones lead to the restricted retrieval of alleles presenting a limitation for phylogenetic analysis. However, due to the genome specific and multi-allelic nature of HMW glutenin gene, the information revealed in this study may potentially contribute to a greater understanding of wheat cultivation in Anatolia almost 8400 years ago. Although several studies have been performed, to date, there is a lack of clarity concerning wheat phylogenetic history due to the unavailability of adequate Triticeae fossils. In an attempt to resolve the polyploidization events, Marcussen, et al. [1] used only single-genome wheat samples (*T*. *urartu* and *Ae*. *tauschii*). Although some relevant information was acquired, the authors suggested that further analyses should include more *T*. *urartu* and *Ae*. *tauschii* progenitors to elucidate the timing and polyploidization events. Hexaploid wheat contains six high molecular weight glutenin subunits (HMW-GS) among which not all are expressed and thus result in the variations in the number of HMW-GSs among the genotypes [48]. Jiang et al. [49] sequenced and characterized both open reading frames (ORFs) and the promoter regions of Glu-1 alleles, and determined low variations among the HMW-GSs of bread wheat. The authors determined that different glutenin subunits have resulted from the duplication of repetitive domains. Our study focused on the evaluation of partial promoter regions of the HMW glutenin genes of different wheat genomes.

It has been well established that einkorn and emmer wheat was first domesticated in the Karacadag region of Diyarbakir in Turkey that is a part of the Fertile Crescent [19]. It is also widely accepted that there is a positive correlation between ploidy levels and the crop development. Polyploid species (representing more than 70% of plant species) acquire more extended geographic distribution than those of their close diploid relatives and this was also evidence for the domestication of wheat. Hence, emmer was the most important crop of Fertile Crescent until the end of Bronze Age. After the replacement of emmer wheat by free-threshing wheat, this trend was followed by the cultivation of bread wheat that expanded more than the durum wheat. It is easy to explain the superiority of tetraploids over diploids; however, it is difficult to understand the reason behind why hexaploid wheat is more robust than the large seeds of tetraploid wheat. This feature can be attributed to allopolyploidy in the hexaploid wheat that gives additional potential to cope with different environmental conditions. After domestication, the subpopulations of emmer wheat diverged following two paths; the southern subpopulation (in Jericho) and the eastern/southeastern subpopulations (through Armenia/Syria and Iraq/Iran). The southern population achieved extended diversity while the eastern subpopulation co-existed with *Ae*. *tauschii* (DD genome progenitor) and led to the development of hexaploid wheat via hybridization. Apparently, the role of the highly developed settlement of that time, Çatalhöyük, was not foreseen. Our molecular evidence suggest that at least in the expansion of the hexaploid wheat cultivation, Çatalhöyük is the center of interest with its crucial position in the development of agriculture and civilization in the western world. Our data shows the existence of higher diversity of HMW glutenin gene promoter regions in the Çatalhöyük samples. We can speculate that hexaploid wheat cultivation had been started in Çatalhöyük before the estimated time for wheat cultivation. Our data is in accord with the most current knowledge concerning the evolution and domestication of wheat and it may contribute to the understanding of the phylogenetic history of wheat.

## Conclusion

In this study, ancient DNA analysis of wheat samples from Near Eastern Turkey was conducted for the first time. The ancient wheat from Çatalhöyük represents the oldest wheat DNA recovered from charred seeds to date and this work provides a new DNA based evidence regarding the species development and wheat evolution. According to the DNA sequence analysis of the 8400-year-old wheat samples, our data provides the first molecular evidence for the expansion of hexaploid wheat cultivation. Our study determined the presence of hexaploid wheat dating back to the seventh millennium BC on the Çatalhöyük site in central Turkey that is located outside the Fertile Crescent. Our results revealed a sequence similarity to the contemporary hexaploid species, including both the naked and hulled forms. In addition, ancient DNA sequences from a later date (2000 BC) from East Anatolia (İmamoğlu Höyük) were found to be predominantly similar to modern naked wheat. This study successfully assessed the genetic similarity of past representatives of cultivated wheat when compared with contemporary wild and cultivated wheat. The results produced novel information on wheat evolution, species formation, domestication and spread. Thus, this study is critically important as being a center of wheat domestication and its spread, the crucial value of Turkey needs to be further investigated.

## Supporting Information

S1 Fig Autoradiograph of radioactively labeled PCR amplification products of Çatalhöyük einkorn samples. Autoradiograph of radioactively labeled PCR amplification products using SP6 and T7 primers from the colonies of the Çatalhöyük einkorn samples (Fig 3) separated on DNA sequencing gel. The colony numbers marked with a circle were selected as representative fragment sizes and those clones were sequenced.
(TIF)

S2 Fig Autoradiograph of radioactively labeled PCR amplification products of Çatalhöyük emmer samples. Autoradiograph of radioactively labeled PCR amplification products using SP6 and T7 primers from the colonies of the Çatalhöyük emmer samples (Fig 3) separated on DNA sequencing gel. The colony numbers marked with a red circle were selected as representative fragment sizes and those clones were sequenced.
(TIF)

S3 Fig Ancient (Çatalhöyük and İmamoğlu H.) wheat sequences at 106–107 bp length. Ancient (Çatalhöyük and İmamoğlu H.) wheat sequences obtained in this study at 106–107 bp length (Total 21 sequences). Alignment was generated by Clustal 1.8 after excising the primer sites and viewed using Chroma software.
(TIF)

S4 Fig Ancient (Çatalhöyük and İmamoğlu Höyük) and modern wheat sequences at 152–156 bp length. Ancient (Çatalhöyük and İmamoğlu Höyük) and modern wheat sequences obtained in this study at 152–156 bp length (Total 43 sequences). Alignment was generated by Clustal 1.8 after excising the primer sites and viewed using Chroma software.
(TIF)

S5 Fig Modern and ancient (İmamoğlu Höyük) wheat sequences at 241–243 bp length. Modern and ancient (İmamoğlu Höyük) wheat sequences obtained in this study at 241–243 bp length (Total 32 sequences). Alignment was generated by Clustal 1.8 after excising the primer sites and viewed using Chroma software.
(TIF)

S6 Fig NJ tree of ancient (Çatalhöyük and İmamoğlu Höyük) wheat sequences at ∼100 bp length. The tree is constructed with ClustalX 1.8 after excising the primer sites with 1000 bootstrap values.
(TIF)

S7 Fig NJ tree of modern and ancient wheat sequences (Çatalhöyük and İmamoğlu Höyük) at 150 bp length. Tree is obtained by ClustalX 1.8 with 1000 bootstrap values after excising the primer sites.
(TIF)

S8 Fig NJ tree of modern and ancient (İmamoğlu Höyük) wheat sequences at ~250 bp length. The tree is constructed with ClustalX 1.8 after excising the primer sites with 1000 bootstrap values.
(TIF)

S9 Fig Genetic relationship of wheat at glutenin loci based on ancient and modern wheat sequences from Turkey. The NJ tree is based on ~100 bp length DNA sequences after excising the primer sites and bootstrapped 1000 times.
(TIF)

S10 Fig Genetic relationship between İmamoğlu Höyük and modern wheat sequences from Turkey and from GenBank at glutenin loci. The NJ tree is based on ~100 bp length DNA sequences after excising the primer sites & bootstrapped 1000 times.
(TIF)

S1 Table Modern wheat samples used for comparison with the ancient wheat DNA analysis. (PDF)

S2 Table Effect of the amount of the starting material in ancient DNA amplifications in relation to the state of preservation and the extraction method. (PDF)

S3 Table Total amplified loci and number of sequences obtained in the study. (PDF)
