# Supplementary figures and images for: Ancient DNA from 8400 Year-Old Çatalhöyük Wheat: Implications for the Origin of Neolithic Agriculture
Source: PLoS One. 2016 Mar 21;11(3):e0151974. doi: 10.1371/journal.pone.0151974 (PMC4801371; doi:10.1371/journal.pone.0151974)

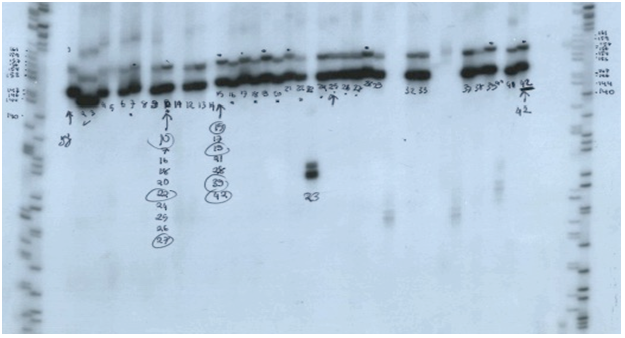

Supplement: S1 Fig — Autoradiograph of radioactively labeled PCR amplification products using SP6 and T7 primers from the colonies of the Çatalhöyük einkorn samples (Fig 3) separated on DNA sequencing gel. The colony numbers marked with a circle were selected as representative fragment sizes and those clones were sequenced. (TIF) [file pone.0151974.s001.tif]

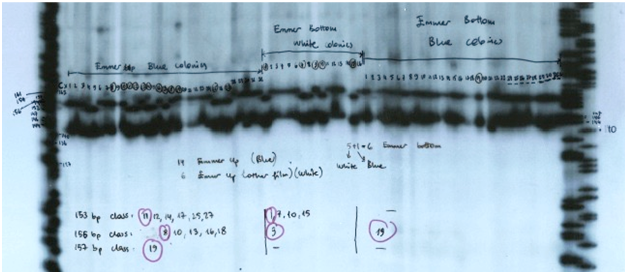

Supplement: S2 Fig — Autoradiograph of radioactively labeled PCR amplification products using SP6 and T7 primers from the colonies of the Çatalhöyük emmer samples (Fig 3) separated on DNA sequencing gel. The colony numbers marked with a red circle were selected as representative fragment sizes and those clones were sequenced. (TIF) [file pone.0151974.s002.tif]

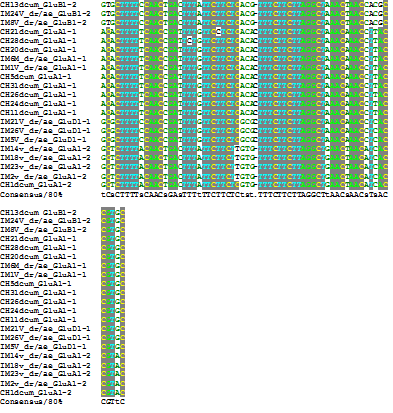

Supplement: S3 Fig — Ancient (Çatalhöyük and İmamoğlu H.) wheat sequences obtained in this study at 106–107 bp length (Total 21 sequences). Alignment was generated by Clustal 1.8 after excising the primer sites and viewed using Chroma software. (TIF) [file pone.0151974.s003.tif]

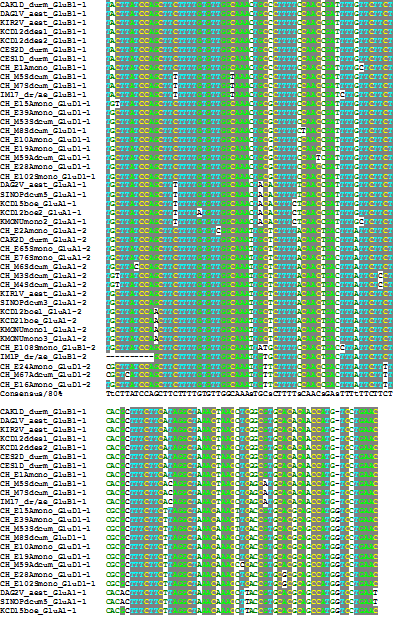

Supplement: S4 Fig — Ancient (Çatalhöyük and İmamoğlu Höyük) and modern wheat sequences obtained in this study at 152–156 bp length (Total 43 sequences). Alignment was generated by Clustal 1.8 after excising the primer sites and viewed using Chroma software. (TIF) [file pone.0151974.s004.tif]

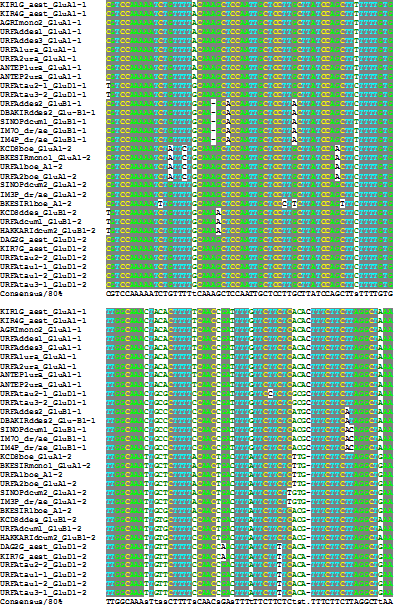

Supplement: S5 Fig — Modern and ancient (İmamoğlu Höyük) wheat sequences obtained in this study at 241–243 bp length (Total 32 sequences). Alignment was generated by Clustal 1.8 after excising the primer sites and viewed using Chroma software. (TIF) [file pone.0151974.s005.tif]

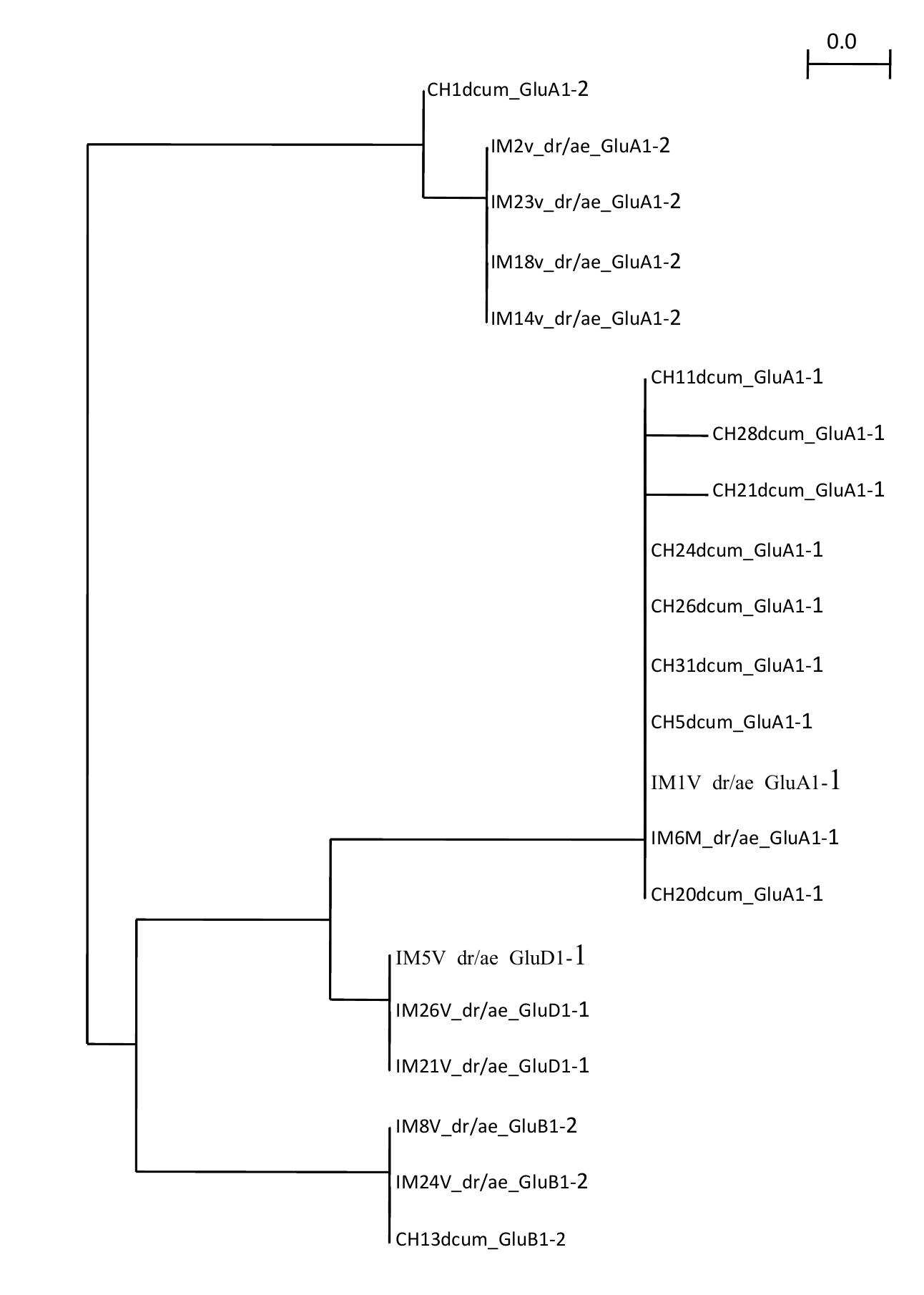

Supplement: S6 Fig — The tree is constructed with ClustalX 1.8 after excising the primer sites with 1000 bootstrap values. (TIF) [file pone.0151974.s006.tif]

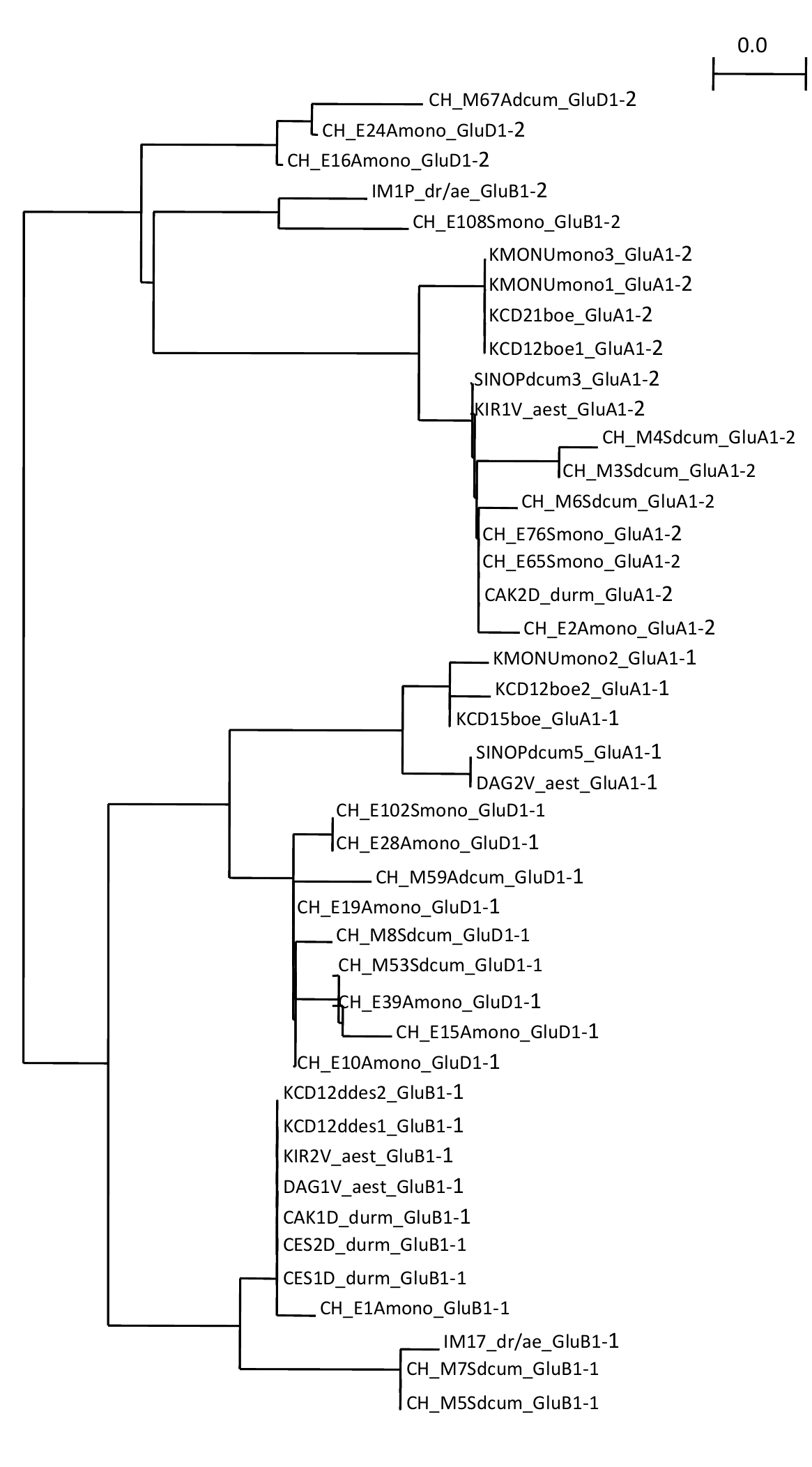

Supplement: S7 Fig — Tree is obtained by ClustalX 1.8 with 1000 bootstrap values after excising the primer sites. (TIF) [file pone.0151974.s007.tif]

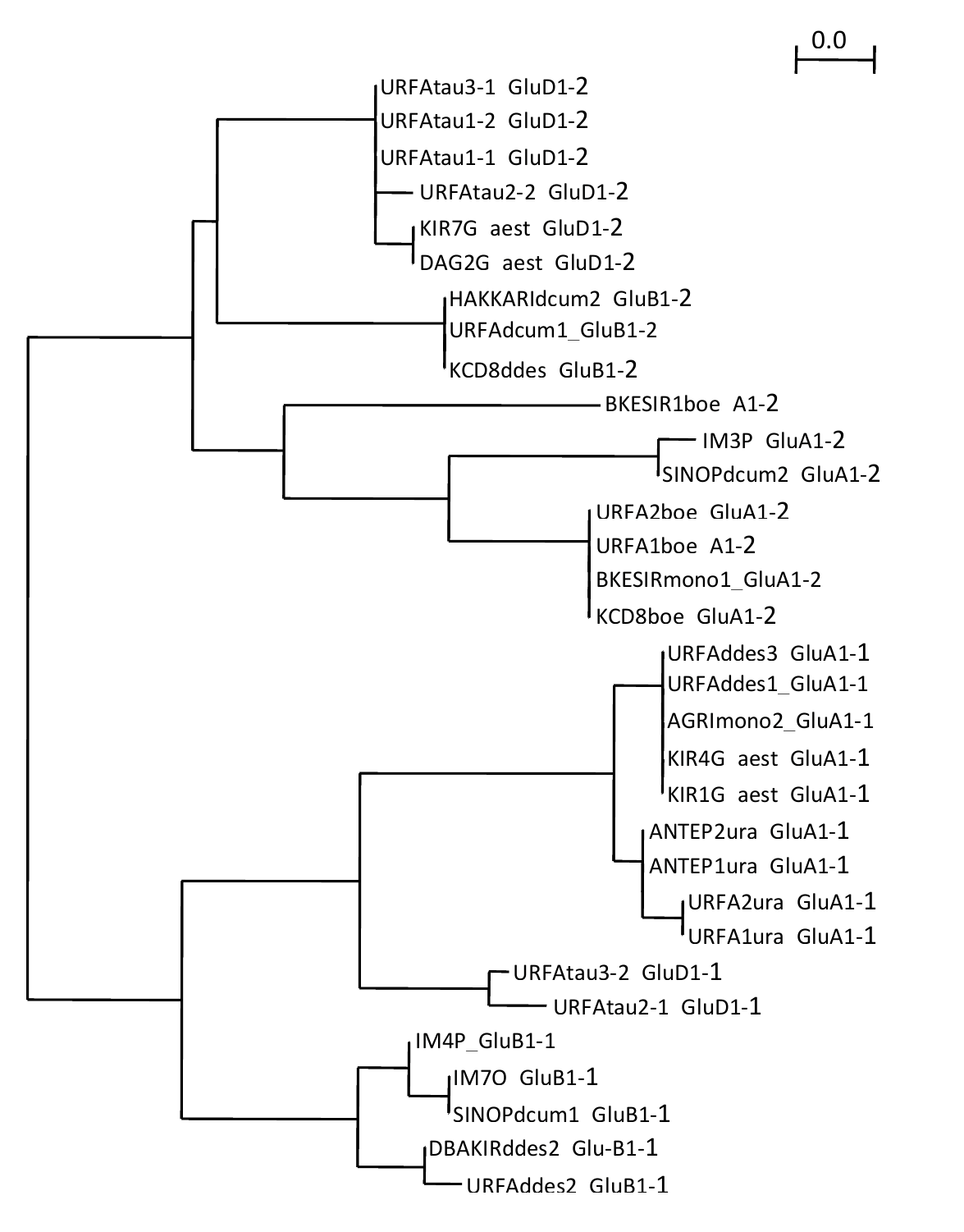

Supplement: S8 Fig — The tree is constructed with ClustalX 1.8 after excising the primer sites with 1000 bootstrap values. (TIF) [file pone.0151974.s008.tif]

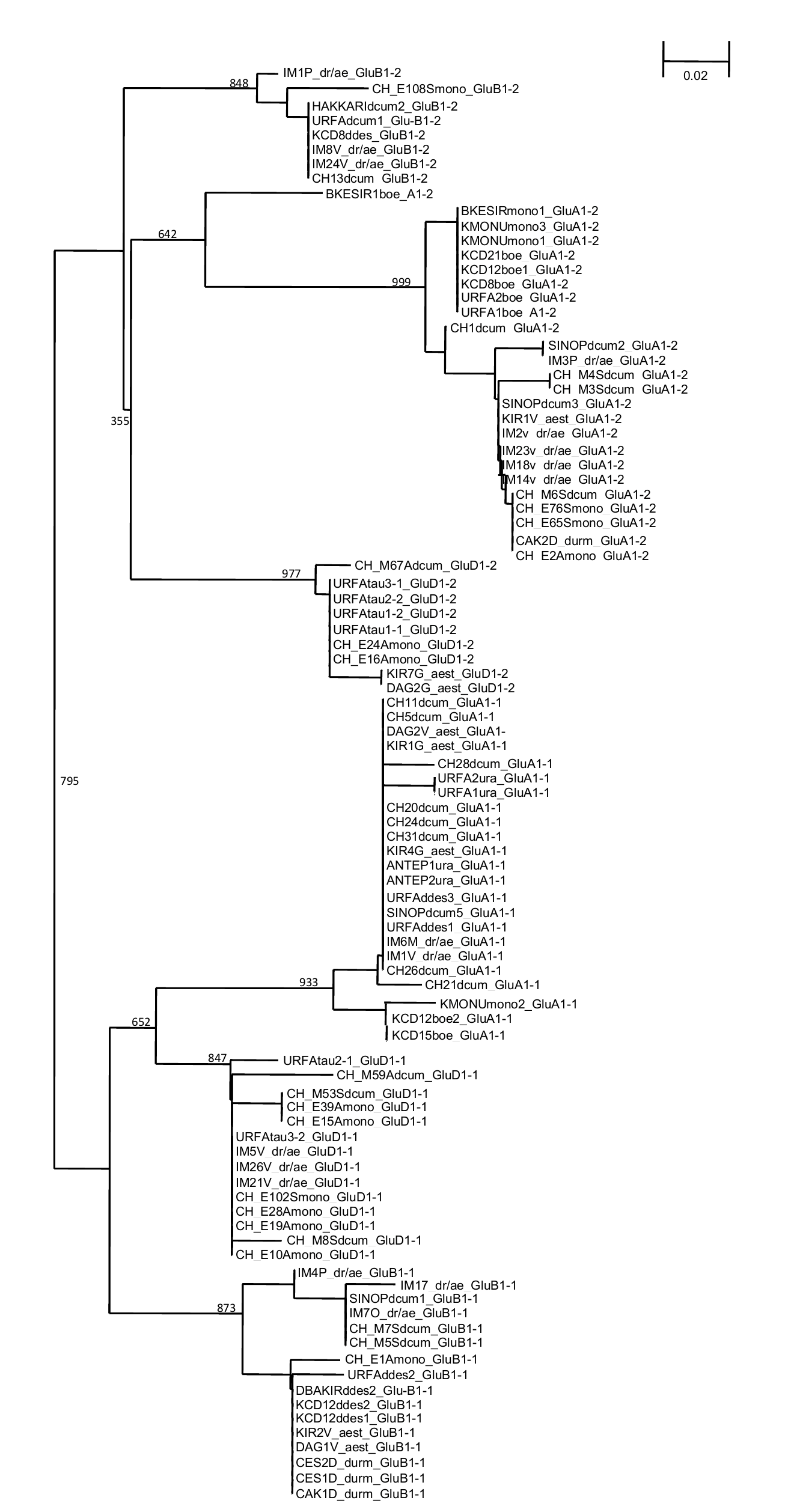

Supplement: S9 Fig — The NJ tree is based on ~100 bp length DNA sequences after excising the primer sites and bootstrapped 1000 times. (TIF) [file pone.0151974.s009.tif]

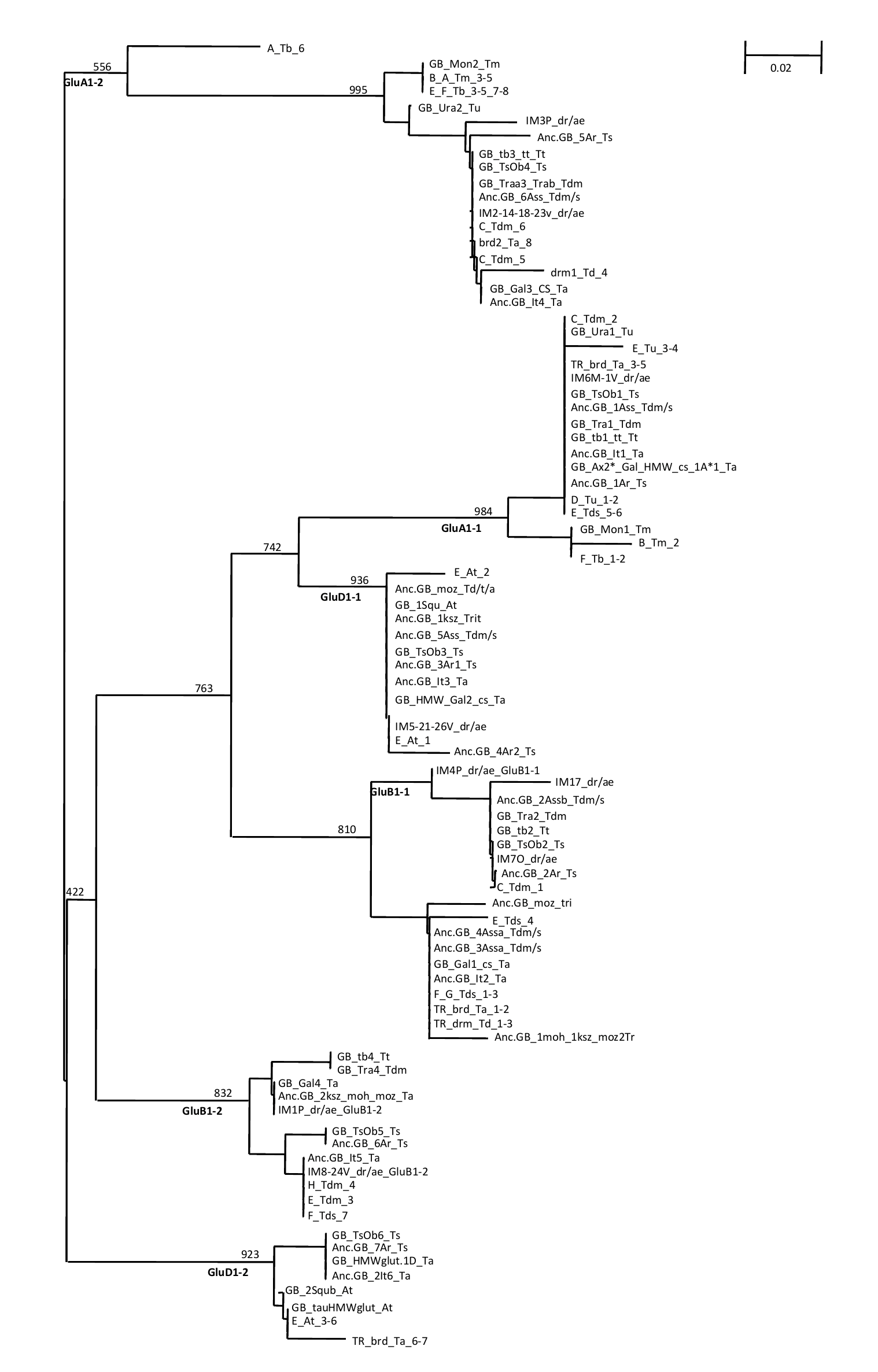

Supplement: S10 Fig — The NJ tree is based on ~100 bp length DNA sequences after excising the primer sites & bootstrapped 1000 times. (TIF) [file pone.0151974.s010.tif]
