# Supplementary material for: Ancient DNA from 8400 Year-Old Çatalhöyük Wheat: Implications for the Origin of Neolithic Agriculture
Source: PLoS One. 2016 Mar 21;11(3):e0151974. doi: 10.1371/journal.pone.0151974 (PMC4801371; doi:10.1371/journal.pone.0151974)
Supplement: S1 Table — (PDF) [file pone.0151974.s011.pdf]

S1 Table. Modern wheat samples included in the ancient wheat DNA analysis for comparison.

Accessions	Species	Code for species	Genome	Origin	Codes for origin
TUR03356	*T. boeoticum*	boe, Tb	AA	Diyarbakır, Karacadağ	F
TUR03363	*T. boeoticum*	boe, Tb	AA	Diyarbakır, Karacadağ	F
TUR03370	*T. boeoticum*	boe, Tb	AA	Diyarbakır, Karacadağ	F
TUR03383	*T. boeoticum*	boe, Tb	AA	Diyarbakır, Karacadağ	F
TR34980	T. boeoticum	boe, Tb	AA	Balıkesir	A
TR46999	*T. boeoticum*	boe, Tb	AA	Şanlıurfa	E
IG116190	*T. urartu*	ura, Tu	AA	Gaziantep	D
IG46113	*T. urartu*	ura, Tu	AA	Şanlıurfa	E
TUR02343	*T. monococccum*	mono, Tm	AA	Kastamonu	B
TR36938	*T. monococccum*	mono, Tm	AA	Balıkesir	A
IG45257	*T. monococccum*	mono, Tm	AA	Ağrı	I
IG45033	*T. dicoccum*	dcum, Tdc	AABB	Hakkari	H
TUR02452	*T. dicoccum*	dcum, Tdm	AABB	Sinop	C
IG44961	*T. dicoccum*	dcum, Tdm	AABB	Şanlıurfa	E
TUR03358	*T. dicoccoides*	ddes, Tds	AABB	Diyarbakır, Karacadağ	F
TUR03362	*T. dicoccoides*	ddes, Tds	AABB	Diyarbakır, Karacadağ	F
IG46253	*T. dicoccoides*	ddes, Tds	AABB	Diyarbakır	G
IG46160	*T. dicoccoides*	ddes, Tds	AABB	Şanlıurfa	E
TUR02628	*T. dicoccoides*	ddes, Tds	AABB	Şanlıurfa, Ceylanpınar	E
TUR02770	*A. tauschii*	tau, At	DD	Şanlıurfa, Ceylanpınar	E
TUR02554	*A. tauschii*	tau, At	DD	Şanlıurfa, Ceylanpınar	E
TUR03500	*A. tauschii*	tau, At	DD	Şanlıurfa, Ceylanpınar	E
Çakmak79	*T. durum*	drm, Td	AABB	Cultivar	-
Çeşit1252	*T. durum*	drm, Td	AABB	Cultivar	-
Dağdaş94	*T. aestivum*	drm, Td	AABBDD	Cultivar	-
Kıraç66	*T. aestivum*	drm, Td	AABBDD	Cultivar	-

Species name and locations were coded to be used in construction of genetic relationship trees.
