# Supplementary material for: Ancient DNA from 8400 Year-Old Çatalhöyük Wheat: Implications for the Origin of Neolithic Agriculture
Source: PLoS One. 2016 Mar 21;11(3):e0151974. doi: 10.1371/journal.pone.0151974 (PMC4801371; doi:10.1371/journal.pone.0151974)
Supplement: S2 Table — (PDF) [file pone.0151974.s012.pdf]

S2 Table. Effect of amount of the starting material in ancient DNA amplifications in relation to the state of preservation and the extraction method.

Sample	Quality	Starting mat.	DNA extraction method	PCR
Çatalhöyük62	UWP	0.5 g	CTAB (EtOH ppt & electroelution)	-
Çatalhöyük61	UWP	0.5 g	CTAB (EtOH ppt & electroelution)	-
Çatalhöyük62	UWP	1.5 g	CTAB (EtOH ppt & electroelution)	+
Çatalhöyük61	UWP	1.5 g	CTAB (EtOH ppt & electroelution)	+
Çatalhöyük61	UWP	0.5 g	CTAB (EtOH ppt only)	+
Çatalhöyük62	UWP	1.0 g	CTAB (No EtOH ppt or electroelution but column purification)	-
Çatalhöyük61	UWP	1.0 g	CTAB (No EtOH ppt or electroelution but column purification)	-
Çatalhöyük62	UWP	1.0 g	CTAB (No EtOH ppt or electroelution but Qiagen purification)	-
Çatalhöyük61	UWP	1.0 g	CTAB (No EtOH ppt or electroelution but Qiagen purification)	-
Çatalhöyük61	UWP	20 seeds	CTAB (No EtOH ppt or electroelution but column purification)	-
Çatalhöyük62	UWP	15 seeds	Direct extraction	-
Çatalhöyük61	UWP	15 seeds	Direct extraction	-
Çatalhöyük61	UWP	Single seed	Single seed extraction	-
Çatalhöyük62	UWP	Single seed	Single seed extraction	-
İmamoğlu H.	WP	0.5 g	CTAB (EtOH ppt & electroelution)	+
İmamoğlu H.	WP	1.5g	CTAB (EtOH ppt only)	+
İmamoğlu H.	WP	0.5 g	CTAB (EtOH ppt only)	+
İmamoğlu H.	WP	Single seed	Single seed extraction	+
İmamoğlu H.	WP	20 seeds	CTAB (No EtOH ppt or electroelution but column purification)	-
İmamoğlu H.	WP	Single seed	Column extraction	-
Patnos (emmer)	WP/b	0.5 g	CTAB (EtOH ppt & electroelution)	+
Patnos (emmer)	WP/b	1.5 g	CTAB (EtOH ppt only)	+
Patnos (naked)	WP/g	0.5 g	CTAB (EtOH ppt & electroelution)	-
Patnos (naked)	WP/g	20 seeds	CTAB (No EtOH ppt or electroelution but column purification)	-
Patnos (emmer)	P/b	0.5 g	CTAB (EtOH ppt & electroelution)	-

UWP: Unusually well preserved; WP: Well preserved; WP/b: Well preserved/black; WP/g: Well preserved/grey; P/b: Powder black
