# Supplementary material for: Ancient DNA from 8400 Year-Old Çatalhöyük Wheat: Implications for the Origin of Neolithic Agriculture
Source: PLoS One. 2016 Mar 21;11(3):e0151974. doi: 10.1371/journal.pone.0151974 (PMC4801371; doi:10.1371/journal.pone.0151974)
Supplement: S3 Table — (PDF) [file pone.0151974.s013.pdf]

S3 Table. Total amplified loci and number of sequences obtained in the study

Sample	Amplified Loci	Number of Sequences Obtained		
		**~250 bp**	**~150 bp**	**~100 bp**
Çatalhöyük	~150 bp, ~100 bp	-	22	10
İmamoğlu Höyük	~250 bp, ~150 bp, ~100 bp	3	2	11
Patnos	~250 bp, ~150 bp	-	-	-
26 modern wheat samples	~250 bp, ~150 bp	29	19	-
